# Molecular Determinants of the Response of Tumor Cells to Boswellic Acids

**DOI:** 10.3390/ph4081171

**Published:** 2011-08-19

**Authors:** Tolga Eichhorn, Henry Johannes Greten, Thomas Efferth

**Affiliations:** 1 Department of Pharmaceutical Biology, Institute of Pharmacy and Biochemistry, Johannes Gutenberg University, Mainz, Staudinger Weg 5, 55128 Mainz, Germany; 2 Heidelberg School of Chinese Medicine, 69126 Heidelberg, Germany

**Keywords:** apoptosis, *Boswellia*, ceramide, frankincense, natural products, olibanum, pharmacogenomics, sphingomyelin

## Abstract

Frankincense (*Boswellia serrata, B. carterii*) is used as traditional remedy to treat inflammatory diseases. The molecular effects of the active ingredients, the boswellic acids, on the immune system have previously been studied and verified in several clinical studies. Boswellic acids also inhibit cancer cell growth *in vitro* and *in vivo*. The molecular basis of the cytotoxicity of boswellic acids is, however, not fully understood as yet. By mRNA-based microarray, COMPARE, and hierarchical cluster analyses, we identified a panel of genes from diverse functional groups, which were significantly associated with sensitivity or resistance of α- or β-boswellic acids, such as transcription factors, signal transducers, growth regulating genes, genes involved in RNA and protein metabolism and others. This indicates that boswellic acids exert profound cytotoxicity on cancer cells by a multiplicity of molecular mechanisms.

## 1. Introduction

*Boswellia serrata* Roxb. et Colebr. and *Boswellia carterii* Birdw. (syn. *B. sacra*) are deciduous trees growing in China, India, the Arab peninsula, and some African countries (Somalia, Ethiopia). The resin gum of these trees is called frankincense or olibanum and is used not only for religious rituals, but also for medicinal purposes in different civilizations [1]. The active principles of frankincense are the boswellic acids, which are pentacyclic triterpenic acids. Since ancient times, frankincense has been used as a remedy to treat inflammatory diseases. The cellular and molecular mechanisms of boswellic acids on the immunological functions in the body have been unraveled since the 1980s and verified in several clinical studies, as recently reviewed [2,3]. Several studies have indicated that boswellic acids also exert growth inhibitory activity towards cancer cells *in vitro* and *in vivo* [4-7].

In recent years, it became evident that molecular mechanisms of inflammatory processes are also relevant for carcinogenesis [8-10]. The connection between inflammation and cancer based on common molecular modes of action raises the question, whether boswellic acids might act against cancer cells by similar mechanisms as those that confer their anti-inflammatory effects. The molecular basis of the cytotoxic action of boswellic acids towards cancer cells is, however, not fully understood as yet.

The aim of the present investigation was, therefore, to analyze the mechanisms of boswellic acids in cancer cells in more detail. For this reason, we were interested to identify possible determinants of sensitivity and resistance of tumor cells towards boswellic acids. We correlated the transcriptomic microarray-based mRNA expression of the cell line panel of the U.S. National Cancer Institute (NCI), with the IC_50_ values for boswellic acids by means of bioinformatic approaches to identify novel molecular determinants for response towards these compounds.

## 2. Experimental Section

### 2.1. Phytochemicals

**Figure 1:**
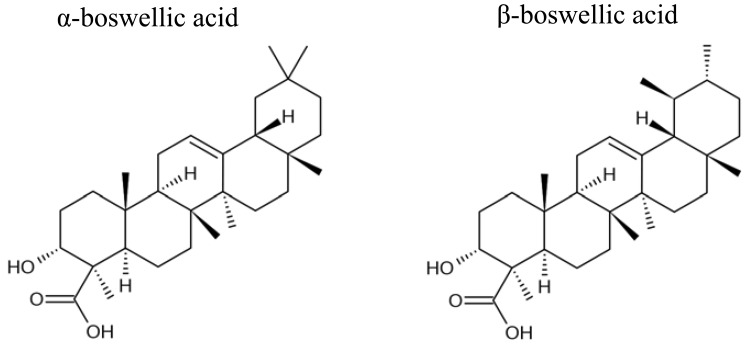
Chemical structures of α- and β-boswellic acid (http://en.wikipedia.org/wiki/Boswellic_acid).

Boswellic acids were obtained from Sigma-Aldrich (Taufkirchen, Germany). The chemical structures are shown in Figure 1.

### 2.2. Cell Lines

The panel of human tumor cell lines of the Developmental Therapeutics Program of NCI consists of leukemia, melanoma, non-small cell lung cancer, colon cancer, renal cancer, ovarian cancer cells, tumor cells of the central nervous system, prostate carcinoma, and breast cancer. Their origin and processing have previously been described [11]. These cell lines were employed to determine the cytotoxicity of α- and β-boswellic acids in comparison to other constituents of *Boswellia carterii* (syn. *B. sacra*) (dipentene, farnesol, and borneol) and to established anticancer drugs (melphalan, teniposide, doxorubicin, vincristine, paclitaxel, and methotrexate).

### 2.3. Sulforhodamine B Assay

The cytotoxicity of phytochemical compounds towards the NCI cell line panel was evaluated by determining the IC_50_ (concentration resulting in 50% inhibition) using a modification of the sulforhodamine B assay [12] (http://dtp.nci.nih.gov/branches/btb/ivclsp.html): Cells were inoculated into 96 well microtiter plates in 100 μL at plating densities ranging from 5,000 to 40,000 cells/well depending on the doubling time of individual cell lines. Microtiter plates were incubated at 37 °C, 5% CO_2_ for 24 h prior to addition of boswellic acids. Then, two plates of each cell line were fixed *in situ* with trichloroacetic acid (TCA) to represent a measurement of the cell population for each cell line at the time of drug addition (Tz). Following addition of boswellic acid, the plates were incubated for an additional 48 h at 37 °C and 5% CO_2_. For adherent cells, the assay is terminated by the addition of 50 μL of cold 50% (w/v) TCA and incubated for 60 min at 4 °C. The supernatant was discarded, and the plates were washed and air dried. Sulforhodamine B (SRB, Sigma, USA;) solution (100 μL) at 0.4% (w/v) in 1% acetic acid was added to each well, and plates were incubated for 10 min at room temperature. Unbound dye was removed by washing with 1% acetic acid and the plates were air dried. Bound stain was subsequently solubilized with 10 mM Trizma base, and the absorbance was read on an automated plate reader at a wavelength of 515 nm. For suspension cells, the methodology was the same except that the assay is terminated by fixing settled cells at the bottom of the wells by gently adding 50 μL of 80% TCA. Using the seven absorbance measurements [time zero, (Tz), control growth, (C), and test growth in the presence of drug at the five concentration levels (Ti)], the percentage growth was calculated as:

$$\begin{array}{c} {}[(\text{Ti-Tz})/(\text{C-Tz})]\times 100\;\text{for concentrations for which}\;\text{Ti}>/=\text{Tz}\\ {}[(\text{Ti-Tz})/\text{Tz}]\times 100\;\text{for concentrations for which}\;\text{Ti}<\text{Tz} \end{array}$$

Growth inhibition of 50% (GI50) was calculated from [(Ti-Tz)/(C-Tz)] × 100 = 50, which was the drug concentration resulting in a 50% reduction in the net protein increase (as measured by SRB staining) in control cells during the drug incubation. The drug concentration resulting in total growth inhibition (TGI) was calculated from Ti = Tz.

### 2.4. Statistical Analyses

The mRNA microarray hybridization of the NCI cell line panel has been described [13,14] and the data has been deposited at the NCI website (http://dtp.nci.nih.gov). For hierarchical cluster analysis, objects were classified by calculation of distances according to the closeness of between-individual distances by means of. All objects were assembled into cluster trees (dendrograms). Previously, cluster models have been validated for gene expression profiling and for approaching molecular pharmacology of cancer [13,15]. Hierarchical cluster analyses applying the WARD method were done with the WinSTAT program (Kalmia, Cambridge, MA, USA). Missing values were automatically omitted by the program, and the closeness of two joined objects was calculated by the number of data points they contained. In order to calculate distances between all variables included in the analysis, the program automatically standardizes the variables by transforming the data with a mean = 0 and a variance = 1.

For COMPARE analysis, the mRNA expression values of genes of interest and IC_50_ values for α- and β-boswellic acids were selected from the NCI database. The mRNA expression has been determined by microarray analyses as reported [13]. COMPARE analyses were performed to produce rank-ordered lists of genes expressed in the NCI cell lines. The methodology has been previously described in detail [16]. Briefly, every gene of the NCI microarray database was ranked for similarity of its mRNA expression to the IC_50_ values for the corresponding compound. To derive COMPARE rankings, a scale index of correlations coefficients (R-values) was created. In the standard COMPARE approach, greater mRNA expression in cell lines correlate with enhanced drug resistance, whereas in reverse COMPARE analyses greater mRNA expression in cell lines indicated drug sensitivity. Pearson's correlation test was used to calculate significance values and rank correlation coefficients as relative measure for the linear dependency of two variables. This test was implemented into the WinSTAT Program (Kalmia).

## 3. Results and Discussion

### 3.1. Cytotoxicity of Boswellic Acids towards a Panel of 60 NCI Cell Lines

**Figure 2:**
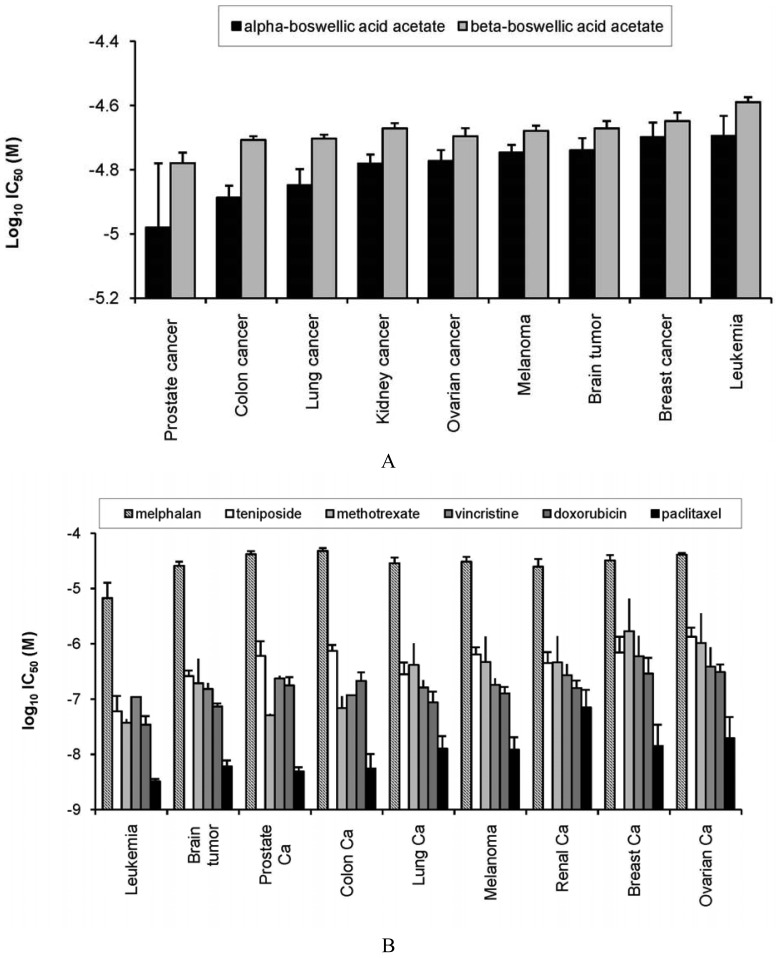
Cytotoxic activity of boswellic acids (A) and established anticancer drugs (B) towards cell lines of different tumor types. 50% inhibition concentration (log_10_ IC_50_) values (M) for α- and β-boswellic acids or standard drugs were determined by the sulforhodamine assay and grouped according to tumor types (mean ± SEM).

As a first step, we investigated the activity of α- and β-boswellic acid towards 60 cell lines of different tumor origin. The IC_50_ values for both compounds have been determined over a dose range of 10^−8^ to 10^−4^ M in the cell line panel and deposited at the database of the NCI's Developmental Therapeutics Program. The log_10_ IC_50_ mean values for these cell lines grouped according to their tumor type are shown in Figure 2A. Across all tumor types, α-boswellic acid was more cytotoxic than β-boswellic acid. Prostate cancer cell lines were most sensitive towards both boswellic acids, whereas breast cancer and leukemia cell lines were most resistant. Cell lines from colon, lung, kidney, or ovarian cancer, melanoma, or brain tumors showed intermediate sensitivity (Figure 2A). The profile of boswellic acids was compared with the response of the cell line panel towards standard anticancer agents (melphalan, teniposide, doxorubicin, vincristine, paclitaxel, and methotrexate). As shown in Figure 2B, two major differences were observed. First, established anticancer drugs inhibited cell lines at lower concentrations (log_10_ IC_50_ values of −8 to −5 M) than boswellic acids (log_10_ IC_50_ values of −5 to −4.5 M). Second, leukemia cell lines were most sensitive to standard anticancer agents, but were most resistant towards boswellic acids. On the other hand, prostate cancer which were most sensitive to boswellic acids, were only intermediate responsive towards standard agents (Figure 2B).

As reported in Duke's Phytochemical and Ethnobotanical Database (http://www.ars-grin.gov/duke/) *Boswellia carterii* (syn. *B. sacra*) contains a number of other phytochemicals in addition to boswellic acids. Among them dipentene, farnesol, and borneol are also deposited in the NCI database. This allowed us to subject the IC_50_ values of α- and β-boswellic acid and those of dipentene, farnesol, and borneol to Pearson's correlation test and to investigate the cross-resistance of cell lines towards these five phytochemicals. Although the correlation of IC_50_ values for α-boswellic acid and those for β-boswellic acid reached a significance level of P < 0.001, the correlation coefficient was rather weak (R < 0.55; Table 1). The IC_50_ values for α-boswellic acid were associated with the IC_50_ values for farnesol and borneol at significance values of P < 0.05, however, the correlation coefficients were weak. Other significant correlations were not found indicating that cross-resistance of these compounds was weakly or not expressed in this panel of cell lines.

### 3.2. mRNA Microarray and COMPARE Analyses

We further investigated the microarray-based transcriptomic mRNA expression by COMPARE analyses to test whether sensitivity and resistance to the boswellic acids were correlated with expression of similar or different sets of genes. We mined the genome-wide mRNA expression database of the NCI and correlated the expression data with the IC_50_ values for α- and β-boswellic acid. This represents a hypothesis-generating bioinformatical approach, which allows the identification of novel putative molecular determinants of cellular response towards arsenic trioxide. First, standard COMPARE analyses were performed. Lowest IC_50_ values of cell lines were correlated with the lowest mRNA expression levels of genes. Then, a reverse COMPARE analysis was done which correlated lowest IC_50_ values with the highest gene expression level. Genes with correlation coefficients of R > 0.55 (standard COMPARE) and R < −0.55 (reverse COMPARE) are listed in Table 2.

Among the genes which associated with cellular response to α-boswellic acid were genes from diverse functional groups such as transcription factors and signal transduction (*PML*, *GLI1*, *PHKA1*), RNA and protein metabolism (*MRPS16*, *FBOX21*, *LSM12*, *SIRT3*) and others (*NEBL*, *TYSND1*, *SLC25A46*). Genes associated with sensitivity or resistance towards β-boswellic acid were also transcriptional factors and signal transducers (*TFCP2, ZNF562, SP7, RAB1, TACC2, TBC1D1*), growth regulators (*PHLDA2, U50277, RPA2, DLGAP5, CEP350*) or others (*SPR, MLL2, SLC25A39, APCS, CYLC1, MYL3*) (Table 2).

**Figure 3:**
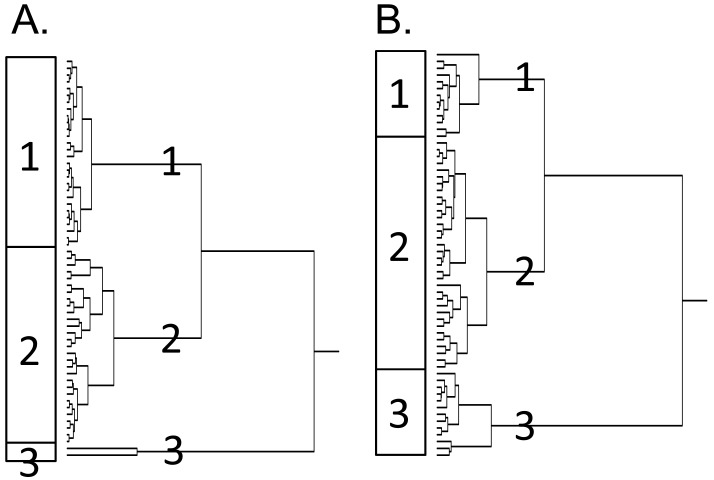
Dendrograms obtained by hierarchical cluster analysis of log_10_ IC_50_ values for (**A**) α-boswellic acid and (**B**) β-boswellic acid of 60 cancer cell lines. The dendrograms were obtained by clustering using the WARD method.

Next, the genes identified by standard and reverse COMPARE analyses were subjected to hierarchical cluster analysis. The dendrograms both for α-boswellic acid (Figure 3A) and β-boswellic acid (Figure 3B) obtained by this procedure can be divided into each three major branches (clusters). To examine whether these clusters were associated with the response to the boswellic acids, these clusters were correlated to the IC_50_ data for these compounds that had not been included before the cluster analysis. Indeed, the distribution of cell lines being sensitive or resistant to the compounds was significantly different between the branches of the dendrograms. The distribution of cell lines among the dendrogram in Figure 3A predicted resistance to α-boswellic acid with significance (P = 4.622 × 10^−7^; χ^2^-test), but not towards β-boswellic acid or other phytochemicals of *Boswellia carterii* (Table 3). Similarly, the distribution of cell lines among the dendrogram in Figure 3B predicted cellular response to β-boswellic acid with significance (P = 1.350 × 10^−5^; χ^2^-test) (Table 4). While sensitivity or resistance to α-boswellic acid was also significantly predicted by this dendrogram, this was not the case for the other constituents of *Boswellia carterii* tested in this investigation (Table 4).

In the present investigation, we analyzed the response of cancer cells towards boswellic acids. A comparison of the IC_50_ values in 60 tumor cell lines showed that boswellic acids inhibited cell lines at higher concentrations than established anticancer drugs. This may indicate that boswellic acids are less effective than classical cytostatic drugs. However, the final efficacy is not only determined by the cytotoxicity of a compound, but also by the concentration window affecting tumor cells in comparison to normal cells. A major disadvantage of most established anticancer drugs is their severe toxicity on normal organs. In contrast, boswellic acids are well tolerated and severe side effects are rare events as pointed out in a recent meta-analysis of clinical trials on *Boswellia* preparations [17].

Another interesting feature of boswellic acids was that prostate cancer cell lines were more sensitive towards these compounds than other cell lines of other tumor types. In many cases, leukemia cells lines are more sensitive to cytotoxic compounds than cell lines from solid cancer types. From these *in vitro* results, it could be speculated that boswellic acids are favorable for the treatment of prostate cancer, albeit clinical experiences are still missing supporting this point of view.

Frankincense gum resin preparations are commercially available rather than isolated boswellic acids. Therefore, the question arises, whether other compounds in addition to α- and β-boswellic acids may also exert cytotoxicity towards cancer cells. While the boswellic derivatives, acetyl-ß-boswellic acid, acetyl-boswellic acid, 11-keto-ß-boswellic acid, and acetyl-11-ß-boswellic acid, are not deposited in the NCI database, the IC_50_ values for dipentene borneol, farnesol were available. Therefore, we have chosen these five phytochemicals to exemplarily analyze cross-resistance among these compounds. Interestingly, we observed that the 60 tumor cell lines did not exert cross-resistance between α- and β-boswellic acid on the one hand and dipentene, borneol, farnesol on the other hand. The development of drug resistance is a tremendous problem in clinical oncology. A general concept of drug resistance has been described by Goldie and Coldman [18]. Starting point of this seminal work were observations with bacterial strains, which acquired resistance towards viruses by spontaneous mutations [19]. Goldie and Coldman and later on other groups developed mathematical models, which explained drug resistance of tumors on the basis of spontaneous mutations of single cells. Upon drug treatment, such resistant cells have a survival advantage compared to the majority of non-mutated sensitive cells and overgrow the entire tumor cell population [20]. Sublethal drug concentrations act as an evolutionary selection pressure for the development of resistant tumors. This can be prevented by the simultaneous treatment with a second drug. The assumption is that small subpopulations resistant to one drug are not resistant at the same time to a second drug. Therefore, they are killed by the second drug and development of resistance to the first drug is avoided. This is the basic principle of combination chemotherapy for tumors developed in the 1970s and 1980s and still well established in clinical oncology up to now. Transferring this concept to medicinal plants, e.g., *B. carterii* provides a similar scenario: small subpopulations resistant to boswellic acids do not survive when they are treated with dipentene, borneol, or farnesol. Hence, boswellic acid resistance of the entire tumor cell population may be avoided. The point of view that phytotherapeutical preparations represent combination therapies, because they contain a multitude of different bioactive phytochemicals, has not been extensively discussed in the literature. Recently, we made comparable observations for different compounds of another medicinal herb, *Artemisia annua* L. [21]. It can be hypothesized that phytochemical preparations with defined contents of phytochemicals may be useful to prevent the emergence of resistance to single compounds.

Furthermore, we analyzed molecular determinants of sensitivity and resistance of cancer tumor cell lines towards α- and β-boswellic acids. By microarray-based gene expression and COMPARE analyses, we correlated the IC_50_ values for both compounds of 60 tumor cell lines with transcriptomic mRNA expression levels of this cell line panel [13]. This approach has been successfully used to unravel the mode of action of novel compounds [22]. Cluster and COMPARE analyses are also useful for comparing gene expression profiles with IC_50_ values for investigational drugs to identify candidate genes for drug resistance [23] and to identify prognostic expression profiles in clinical oncology [24].

We identified genes from diverse functional groups, which were tightly associated with the response of tumor cells to boswellic acids such as transcription factors and signal transducers, growth regulating genes, genes involved in RNA and protein metabolism and others. Although these genes have not yet been associated with cellular response to boswellic acids, the results can be reconciled with a proposed role of boswellic acid in a growth inhibitory activity towards cancer cells. The gene-hunting approach applied by us delivered several novel candidate genes that may regulate the response of cancer cells to boswellic acids. These results merit further investigation to prove the contribution of these genes to boswellic acid resistance. Remarkably, inflammation-related genes did not appear in our analysis. Cancer development is frequently preceded by inflammatory processes [8-10], and boswellic acids are known for their anti-inflammatory activity [2,3]. The fact that inflammation-related genes did not appear in our microarray-based COMPARE analysis indicates that the activity of boswellic acids towards cancer cells might primarily not be linked to inflammation-related mechanisms.

## 4. Conclusions

The fact that genes associated with sensitivity or resistance against α- and β-boswellic acid were from diverse functional groups speaks for the multiplicity of mechanisms whereby boswellic acids exert their inhibitory effects towards cancer cells. Multiplicity of mechanisms can mean that boswellic acids either have multiple targets leading to multiple effects, or one target leading to activation or inactivation of multiple mechanisms downstream of this target. A general feature of natural products is their multi-specificity. Rather than acting on one single target, multiple targets and pathways are affected [25]. Multi-specificity prevents the development of resistance towards one bioactive compounds which turned out to be an important selection advantage during evolution of life [25].

## Figures and Tables

**Table 1:** Cross-resistance profile of a panel od cell lines towards five phytochemicals from *Boswellia carterii* determined by correlating the IC_50_ values by Pearson's correlation test.

		**β-Boswellic acid**	**Dipentene**	**Farnesol**	**Borneol**
**α-Boswellic acid**	R-Value	0.412	−0.085	−0.303	0.276
	P-Value	2.11 × 10^−4^	0.261	0.015	0.021
**β-Boswellic acid**	R-Value		−0.061	−0.219	0.096
	P-Value		0.324	0.058	0.242
**Dipentene**	R-Value			−0.017	0.056
	P-Value			0.455	0.358
**Farnesol**	R-Value				−0.169
	P-Value				0.122

**Table 2:** Genes identified by standard or reverse COMPARE analyses, whose mRNA expression in a panel of 60 cell lines correlated with IC_50_ values for α- and β-boswellic acids.

**COMPARE coefficient**	**Genebank Acc No.**	**Symbol**	**Name**	**Function**
**α-boswellic acid**				
**Standard COMPARE:**				
0.628	BE965646	unknown	unknown	unknown
0.599	NM_006393	*NEBL*	Nebulette	structural constituent of muscle
0.593	H18472	*TYSND1*	Trypsin domain containing 1	peroxisome enzyme
0.592	X73874	*PHKA1*	Phosphorylase kinase, alpha 1 (muscle)	phosphorylase kinase of troponin I, binds calmodulin
0.586	AI806379	*LSM12*	LSM12 homolog (S. cerevisiae)	protein binding
0.582	AB020682	*FBOX21*	F-box protein 21	ubiquitin-protein ligase
0.576	NM_016065	*MRPS16*	Mitochondrial ribosomal protein S16	structural constituent of ribosome
0.576	AI870951	C17orf96	Chromosome 17 open reading frame 96	unknown
0.571	NM_005269	*GLI1*	GLI family zinc finger 1	RNA polymerase II transcription factor	
0.569	AF083108	*SIRT3*	Sirtuin 3	NAD-dependent protein deacetylase	
**Reverse COMPARE:**					
−0.522	R55296	*PML*	Promyelocytic leukemia	transcription factor	
−0.509	N70280	C2orf60	Chromosome 2 open reading frame 60	unknown	
−0.509	R78631	*SLC25A46*	Solute carrier family 25, member 46	transmembrane transport	
**β-boswellic acid**					
**Standard COMPARE:**					
0.661	M76231	*SPR*	Sepiapterin reductase (7,8-dihydrobiopterin:NADP+ oxidoreductase) Myeloid/lymphoid or mixed-lineage	oxidoreductase in tetra-hydrobiopterin biosynthesis	
0.574	AF010403	*MLL2*	leukemia 2	Histone methyltransferase	
0.567	AF097738	unknown	unknown	unknown	
0.563	AI354351	*SLC25A39*	Transcribed locus, similar to NP_057100.1 solute carrier family 25 member 39 isoform b	unknown	
0.558	U03494	*TFCP2*	Transcription factor CP2		
0.545	AF035444	*PHLDA2*	Pleckstrin homology-like domain, family A, member 2	placenta growth regulation	
0.54	H79005	*ZNF652*	Zinc finger protein 652	transcriptional repressor	
0.534	NM_001639	*APCS*	Amyloid P component, serum	sugar, metal, and protein binding protein	
0.534	M28209	*RAB1A*	RAB1A, member RAS oncogene family	GTPase	
0.533	AF220152	*TACC2*	Transforming, acidic coiled-coil containing protein 2	nuclear hormone receptor	
**Reverse COMPARE:**					
−0.588	U50277	unknown	Breast cancer suppressor element Ishmael Upper CP1	tumor suppressor?	
−0.576	W27118	*RPA2*	Replication protein A2, 32kDa	replication and DNA repair	
−0.574	D13633	*DLGAP5*	Discs, large (*Drosophila*) homolog-associated protein 5	cell cycle regulator in carcinogeneisis	
−0.565	W28183	C16orf80	Chromosome 16 open reading frame 80	unknown	
−0.555	AW020776	*SP7*	Sp7 transcription factor	transcription factor	
−0.553	Z22780	*CYLC1*	Cylicin, basic protein of sperm head cytoskeleton 1	structural molecule	
−0.553	NM_014810	*CEP350*	Centrosomal protein 350kDa	centriole growth regulator	
−0.549	AI061288	unknown	unknown	unknown	
−0.544	N92340	*MYL3*	Myosin, light chain 3, alkali; ventricular, skeletal, slow	structural constituent of muscle	
−0.543	AW043925	*TBC1D1*	TBC1 (tre-2/USP6, BUB2, cdc16) domain family, member 1	Rab GTPase activator	

Information on gene functions was taken from the OMIM database, NCI, USA (http://www.ncbi.nlm.nih.gov/Omim/) and from the GeneCard database of the Weizman Institute of Science, Rehovot, Israel. http://bioinfo.weizmann.ac.il/cards/index.html).

**Table 3:** Separation of clusters of 60 cancer cell lines obtained by hierarchical cluster analysis for α-boswellic acid shown in Figures 3A in comparison to other phytochemical constituents of *Boswellia carterii*. The log_10_ IC_50_ median values (M) of each compound were used as cut-off values to define cell lines as being sensitive or resistant. P > 0.05 was considered as not significant (χ^2^ test).

		**Partition**	**Cluster 1**	**Cluster 2**	**Cluster 3**	**χ^2^ Test**
α-Boswellic acid	sensitive	< −4.780	3	23	2	
	resistant	≥ −4.780	25	6	0	P = 4.622 × 10^−7^
β-Boswellic acid	sensitive	< −4.676	11	16	2	
	resistant	≥ −4.676	17	13	0	P = 0.167
Dipentene	sensitive	< −3.036	15	9	0	
	resistant	≥ −3.036	8	16	1	P = 0.079
Farnesol	sensitive	< −4.646	13	10	1	
	resistant	≥ −4.646	10	16	1	P = 0.448
Borneol	sensitive	< −4.0	8	7	1	
	resistant	≥ −4.0	18	20	0	P = 0.277

**Table 4:** Separation of clusters of 60 cell lines obtained by hierarchical cluster analysis for β-boswellic acid shown in Figures 3B in comparison to other phytochemical constituents of *Boswellia carterii*. The log_10_ IC_50_ median values (M) of each compound were used as cut-off values to define cell lines as being sensitive or resistant. P > 0.05 was considered as not significant (χ^2^ test).

		**Partition**	**Cluster 1**	**Cluster 2**	**Cluster 3**	**χ^2^ Test**
β-Boswellic acid	sensitive	< −4.676	12	18	0	
	resistant	≥ −4.676	1	16	13	P = 1.350 × 10^−5^
α-Boswellic acid	sensitive	< −4.780	8	19	1	
	resistant	≥ −4.780	5	14	12	P = 0.005
Dipentene	sensitive	< −3.036	4	13	7	
	resistant	≥ −3.036	7	14	4	P = 0.437
Farnesol	sensitive	< −4.646	2	15	8	
	resistant	≥ −4.646	8	16	3	P = 0.054
Borneol	sensitive	< −4.0	5	5	5	
	resistant	≥ −4.0	8	24	7	P = 0.174
